# Effects of an Exhaustive Exercise on Motor Skill Learning and on the Excitability of Primary Motor Cortex and Supplementary Motor Area

**DOI:** 10.1097/MD.0000000000002978

**Published:** 2016-03-18

**Authors:** Marinella Coco, Vincenzo Perciavalle, Paolo Cavallari, Valentina Perciavalle

**Affiliations:** From the Section of Physiology of the Department of Biomedical and Biotechnological Sciences, Universita‘ degli Studi di Catania, Catania, Italy (MC, ViP); Section of Human Physiology of the Department of Pathophysiology and Transplantation, Universita‘ degli Studi di Milano, Milan, Italy (PC); and Department of Sciences of Formation, Universita‘ degli Studi di Catania, Catania, Italy (VaP).

## Abstract

We examined, on 28 healthy adult subjects, the possible correlations of an exhaustive exercise, and the consequent high blood lactate levels, on immediate (explicit) and delayed (implicit) motor execution of sequential finger movements (cognitive task). Moreover, we determined with transcranial magnetic stimulation whether changes in motor performance are associated with variations in excitability of primary motor area (M1) and supplementary motor area (SMA). We observed that, after an acute exhaustive exercise, the large increase of blood lactate is associated with a significant worsening of both explicit and implicit sequential visuomotor task paradigms, without gender differences. We also found that, at the end of the exhaustive exercise, there is a change of excitability in both M1 and SMA. In particular, the excitability of M1 was increased whereas that of SMA decreased and, also in this case, without gender differences. These results support the idea that an increase of blood lactate after an exhaustive exercise appears to have a protective effect at level of primary cortical areas (as M1), although at the expense of efficiency of adjacent cortical regions (as SMA).

## INTRODUCTION

An exhaustive exercise induces a transient muscle's inability to maintain an optimal performance, called fatigue, dependent on to the occurrence of several factors, having both peripheral and central origins.^1^ In fact, after exhaustive exercise a reduction in muscle phosphocreatine and Adenosine TriPhosphate, as well as an increase of pyruvate and lactate,^2^ with the latter released into the blood,^3^ has been observed. As oxygen, glucose, and lactate extraction by the Central Nervous System increases during maximal exercise, it has been hypothesized that brain activation is enhanced during this behavior.^4,5^ It has also been suggested that in the brain, in particular in conditions of altered energy production, such as anoxia or hypoglycemia, lactate is the energetic metabolite used for sustaining glutamatergic synaptic activity.^6^ However, it has been observed that an increase of blood lactate, induced by an exhaustive exercise or an intravenous infusion, is associated with a worsening of attentional processes,^7^ an improvement of excitability of primary motor cortex,^8^ without significant modifications of spinal^9^ and brainstem^10^ excitability. Furthermore, experiment carried out with visual-evoked potentials^11^ and somatosensory-evoked potentials^12^ have shown that increases of blood lactate are associated, in both cases, with that an improvement in the conduction time between the periphery and the primary sensory areas and the worsening of intracortical communication between primary areas and additional cortical regions. Moreover, different effects induced by lactate have been observed in vitro on neurons and astrocytes.^13^

Primary motor cortex (M1) might contribute to motor learning by optimizing the timing of visuomotor processing.^14^ In fact, activation of M1 seems to produce a task-dependent effect on learning and memory formation^15^ and it appears to be involved in the early consolidation of motor skills.^16^ Instead, the supplementary motor area (SMA) might contribute to the preparation and execution of learned motor sequences;^17,18^ in this way, SMA seems to play an important role in linking cognition to action.^19^ A TMS study showed the existence of connections between M1 and SMA, mediated via excitatory interneurons.^20^ Gerloff et al^21^ observed that 20 Hz repetitive TMS over the SMA induced AC errors in complex sequential movements. This finding indicated a critical role of SMA in the organization of forthcoming movements in complex motor sequences that are drawn from memory and distributed into a precise timing plan. Several studies have also demonstrated that M1 and SMA all appear to be particularly important in the early stage of motor skill acquisition.^22,23^ Recently, Kim and Shin^24^ compared the effects of 20 Hz repetitive TMS on M1 and SMA, in explicit as well as implicit learning of motor skills. Their finding indicated an important role of SMA compared to M1, in implicit motor learning.

Motor skills, as playing piano, require rapid visuomotor coordination and precise sequences of finger movements. These motor skills can be learned either explicitly, practicing after reading the sheet music, or implicitly, after memorizing the piece of music and then self-initiating its execution. Motor performance can be measured by visuomotor response time (RT), task duration (TD), and accuracy (AC), as recently carried out by Kim and Shin.^24^ Thus, the aim of the present study was to examine the possible correlations of an exhaustive exercise, and the consequent high blood lactate levels, on motor learning of sequential finger movements (cognitive task). The visuomotor skills were evaluated by the standards of motor performance which are RT, TD, and AC. Moreover, we determined whether changes in motor skills were associated with variations in excitability of M1 and SMA, as measured by recruitment curve.

## MATERIALS AND METHODS

### Participants

The subjects who volunteered for this study were 28 healthy adults, aging between 27 and 51 years. On the basis of their physical activity, all volunteers can be considered sedentary subjects. Out of these, 14 were women, aging between 27 and 45 years (mean age 35.7 ± 5.51 SD), with a mean height of 167.8 cm ( ± 4.99 SD) and a mean weight of 60.0 kg ( ± 4.99 SD). All the women who participated in the study had a regular menstrual cycle. The remaining 14 volunteers were men aging between 30 and 51 years (mean age 39.1 ± 6.02 SD), with a mean height of 174.1 cm ( ± 4.46 SD) and a mean weight of 68.6 kg ( ± 5.09 SD). None of the volunteers was using drugs capable of changing cortical excitability and only 3 of them were smokers, a man and 2 women. Volunteers gave informed consent to procedures approved by the Ethical Committee of our Universities, which were conducted in accordance with the Declaration of Helsinki.

The volunteers were all right handed according to the Edinburgh handedness inventory.^25^ The subjects were required to have previously performed a structural brain magnetic resonance imaging (MRI) exam with a 1.5 T scanner having contiguous sagittal slices and full brain coverage. In most cases (22 on 28), the examination was carried out to determine the causes of a headache, whereas in the remaining 6 cases was performed after a head injury. In any case, only those subjects whose MRI exam was negative for neurological diseases were included in the present study. Participation criteria included a normal neurological examination, no contraindication for TMS and exhaustive exercise, not being an active musician, and the ability to perform and learn the cognitive tasks.

### Explicit and Implicit Motor Trials

**FIGURE 1:**
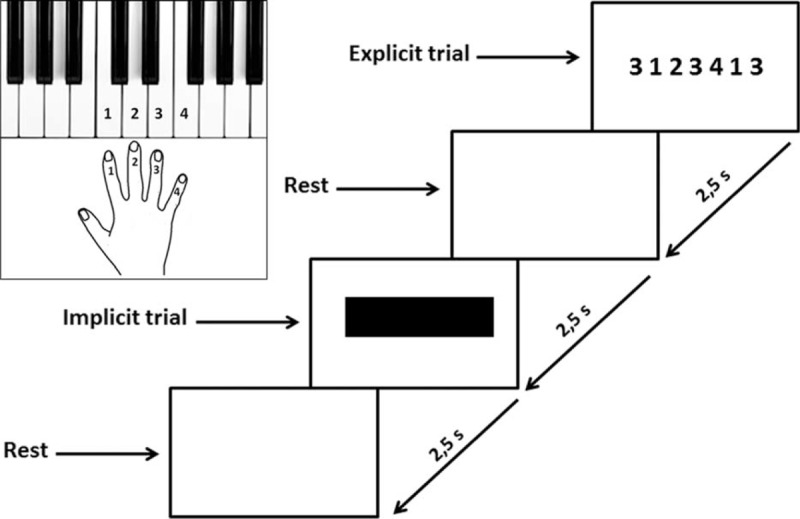
Experimental design for cognitive task. When a 7-digit number stimulus appeared on a computer screen subjects had to press the keyboard keys. The 7-digit sequence of numbers was a combination of 1, 2, 3, or 4 randomly ordered and was displayed on the monitor for 2.5 s (s). Participants were instructed to press the 1, 2, 3, or 4 numbered keys as accurately and quickly as possible, with their right hand. Each key was labeled with a number representing the finger to be used: 1, 2, 3, and 4 represented the index, the middle, the ring, and the little finger, respectively. When the 7-digit number stimulus appeared, subjects had to hit the 1st number as quickly as possible and press the remaining 6 numbered keys for 2.5 s. That was defined as the explicit trial. After a 2.5 s rest, a black bar was displayed on the monitor and the participants had to press the 7-digit sequence of numbers as quickly and accurately as possible on the basis of memory for 2.5 s. That was defined as the implicit trial. It was followed by a rest for 2.5. This 2.5 s × 4 cycle was repeated 4 times. A 20 s rest followed. This entire set was repeated 5 times for a total duration of the cognitive task of 5 min.

The cognitive task was designed by adapting the process recently used by Kim and Shin.^24^ Briefly, volunteers performed a block of sequential finger-tapping tasks. The sequential visuomotor task paradigm required to press the keyboard keys in response to a 7-digit number stimulus appeared on a computer screen (Figure 1). The 7-digit sequence was a combination of 1, 2, 3, or 4 randomly ordered and was displayed on the monitor for 2.5 seconds. Participants were instructed to press the 1, 2, 3, or 4 numbered keys as accurately and quickly as possible, with their right-hand index, the middle, the ring, and the little finger, respectively.

When the 7-digit number stimulus appeared, subjects had to hit the 1st number as quickly as possible and press the remaining 6 numbered keys within 2.5 seconds. That was defined as the explicit trial. After a 2.5 seconds rest, a black bar on the monitor was displayed and the participants had to press the 7-digit sequence of numbers as quickly and accurately as possible on the basis of memory within 2.5 seconds. That was defined as the implicit trial. It was followed by a rest for 2.5 seconds. This 2.5 seconds × 4 cycle was repeated 4 times. A 20 seconds rest followed. This entire set was repeated 5 times for a total duration of the cognitive task of 5 minutes.

The motor performance was determined by assessing RT, TD, and AC using a keyboard Musical Instrument Digital Interface (MIDI) program. RT was the time interval, expressed in millisecond (ms), between the appearance of the 7-digit sequence of numbers and pressing the first key. TD was the time interval, expressed in ms, between the start of pressing the first key and the end of pressing the last key. AC was the total number of correctly pressed key.

### Evaluation of Excitability of M1 and SMA: Motor Threshold and Recruitment Curve

A neuronavigation system (Softaxic Optic system 2.0, E.M.S. srl Company, Bologna, Italy) with an optical tracking system NDI Polaris Vicra (NDI International, Waterloo, Ontario, Canada) was used to maintain constant coil-positioning throughout the entire experiment; MRI data provided by each subject were elaborated to obtain the volumetric reconstruction of individual brain and cortical folders in particular.

The participants were seated at cycloergometer (see below) with the back leaning against a vertical support and electromyographic (EMG) activity was recorded using pairs of silver chloride (AgCl) electrodes. EMG signals were amplified (gain = 1000), bandpass filtered (20 or 30–1000 Hz), and digitized at a sampling rate of 4 kHz.

Magnetic stimuli were delivered by a Magstim 200 stimulator (Magstim Co., Dyfed, UK) using a figure of 8 coil (internal wing diameter 70 mm), to the left M1 located at the optimal position (hot spot) to obtain a motor-evoked potential (MEP) from the first dorsal interosseus (FDI) muscle of the contralateral (right) arm. The coil was placed so that the axis of intersection between the 2 loops was orientated at ∼45 deg to the sagittal plane, to induce posterior to anterior current flow across the motor strip. Once the hot spot was established, the lowest stimulation intensity at which MEPs with peak-to-peak amplitude of ∼50 μV were evoked in at least 5 of 10 consecutive trials was taken as resting motor threshold (RMT).

Previously described criteria were used to determine the site for SMA stimulation^26,27^ that is, the optimal position for activation of the right tibialis anterior (TA) muscle, by moving the coil in 1 cm incremental steps along the midline around the scalp vertex (Cz) with the handle pointing 90° to the left. The active motor threshold (AMT) was determined as the lowest stimulation intensity required to evoke an MEP > 200 μV in 5 out of 10 trials at 20% of maximal voluntary contraction (MVC) of TA. Stimuli of ∼1.3 AMT were given by moving the coil anteriorly along the midline in1 cm steps. The SMA was defined as being 1 cm anterior to the last site from which MEPs could be evoked during the contraction.^28^

The MEP recruitment curve was obtained by delivering TMS at 10% increments of intensity between 100% and 140% of the RMT or AMT, respectively. Six stimuli were delivered at each level of intensity. The order of delivery was randomized. The interval between successive stimuli varied between 4 and 6 seconds. The total duration of the sequence was ∼5 minutes. The average MEP amplitudes obtained at 100% RMT or AMT were calculated to ensure that the threshold had been correctly determined. Equivalent sets of stimuli were delivered immediately following the end of the acute exhaustive exercise.

### Acute Exhaustive Exercise

The participants performed a maximal multistage discontinuous incremental cycling test on a mechanically braked cycloergometer (Monark, Sweden), at a pedaling rate of 60 rpm, whereas an electrocardiogram was monitored. Each subject started with unloaded cycling duration of 3 minutes, and the load was increased by 30 Watt (W) every 3 minutes, until volitional exhaustion or the required pedaling frequency of 60 rpm could not be maintained.^29^

Venous blood lactate levels and venous blood glucose levels were measured before, at the end as well as 7 and 15 minutes after the conclusion of the exhaustive exercise. The blood lactate level was measured by using a “Lactate Pro” portable lactate analyzer (FaCT Canada Consulting Ltd), whereas blood glucose was measured with an “Ascensia Elite” portable blood glucose monitoring system (Bayer AG, Germany).

### Experimental Design

**FIGURE 2:**
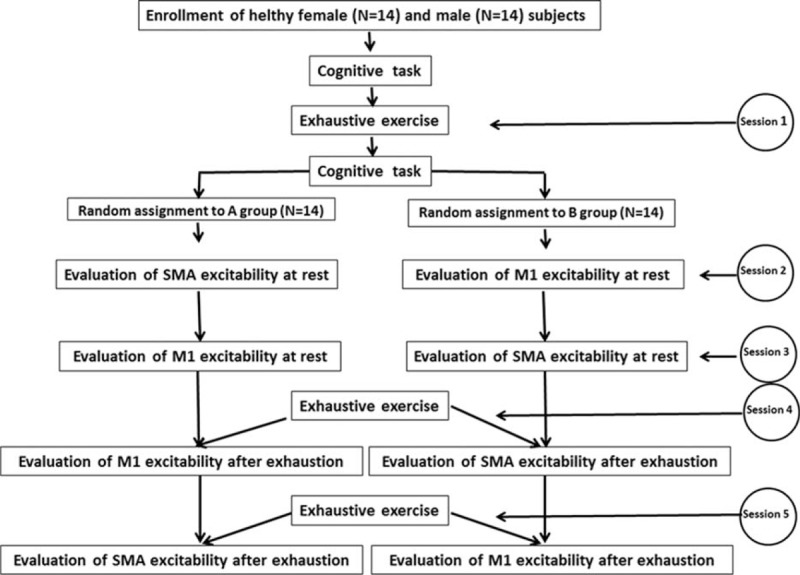
Flowchart illustrating the principal phases of the study.

All experiments were performed between 9 am and 1 pm, with volunteers who had a breakfast before 8 am. As can be seen in Figure 2, each subject had to participate in 5 different experimental sessions:

1. Session 1: each subject performed the cognitive task at rest and blood lactate was measured (before); each subject executed the acute exhaustive exercise and, at the end, executed the cognitive task; blood lactate was measured at the end of the exhaustive exercise, as well as after 7 and 15 minutes.
2. Session 2 at least 4 days after session 1: each subject underwent to the evaluation at rest of M1 excitability or that of SMA (14 subjects for each test, randomly selected).
3. Session 3 at least 7 days after session 2: each subject underwent to the evaluation at rest of the other cortical area.
4. Session 4 at least 7 days after session 3: each subject executed for the second time the exhaustive exercise and, at the end of exercise, underwent to the evaluation of M1 excitability or that of SMA (14 subjects for each test, randomly selected); blood lactate was measured at the end of the exhaustive exercise, as well as after 7 and 15 minutes.
5. Session 5 at least 7 days after session 4: each subject executed for the third time the exhaustive exercise and was submitted to evaluation of cortical excitability at the end of exercise (in each subject was assessed the excitability of cortical area unexamined a week before); blood lactate was measured at the end of the exhaustive exercise, as well as after 7 and 15 minutes.

### Statistical Analysis

Data was collected and averaged, and then compared with the paired *t* test (2-tailed) or 1-way repeated measures analysis of variance (ANOVA; Friedman test), followed by Dunn's Multiple Comparison Test. Significance was set at *P* < 0.05. All descriptive statistics are reported as mean ± SD. All analyses were performed by using GraphPad Prism version 6.03 for Windows (GraphPad Software, San Diego, CA).

## RESULTS

**FIGURE 3:**
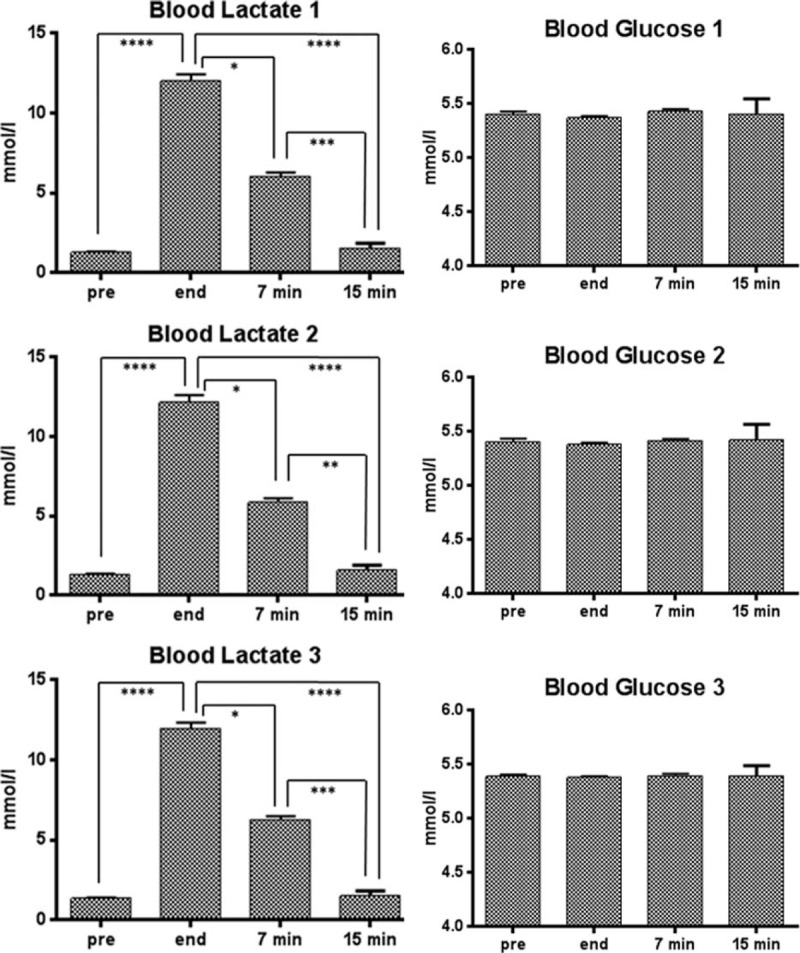
Blood lactate and blood glucose levels ( ± SD) of the 28 subjects performing the acute exhaustive exercise in 3 different days. Lactate and glucose were measured before the exercise (pre), at its conclusion (end), as well as 7 and 15 min after its conclusion. The error bars indicate standard deviation. Symbols from ANOVA with Dunns's Multiple Comparison Test: ^∗^, *P* < 0.05; ^∗∗^, *P* < 0.01; ^∗∗∗^, *P* < 0.001; ^∗∗∗∗^, *P* < 0.0001. SD = standard deviation.

The experimental design required each subject to perform the acute exhaustive exercise in 3 different days, delayed each one of at least 1 week. As can be seen in Figure 3, after the exhaustive exercise, blood lactate levels increased from a mean value of 1.3 to 1.4 mmol/L to a mean value 12.0 to 12.2 mmol/L. Seven minutes after the conclusion of the exercise, the blood lactate levels remained significantly higher than those measured before exercise (mean value of 5.9–6.3 mmol/L) and then returned to pre-exercise values after 15 minutes (mean value of 1.5–1.6 mmol/L), without significant differences in the 3 sessions. Blood glucose levels, instead, did not show significant changes in relation to the exhaustive exercise.

**FIGURE 4:**
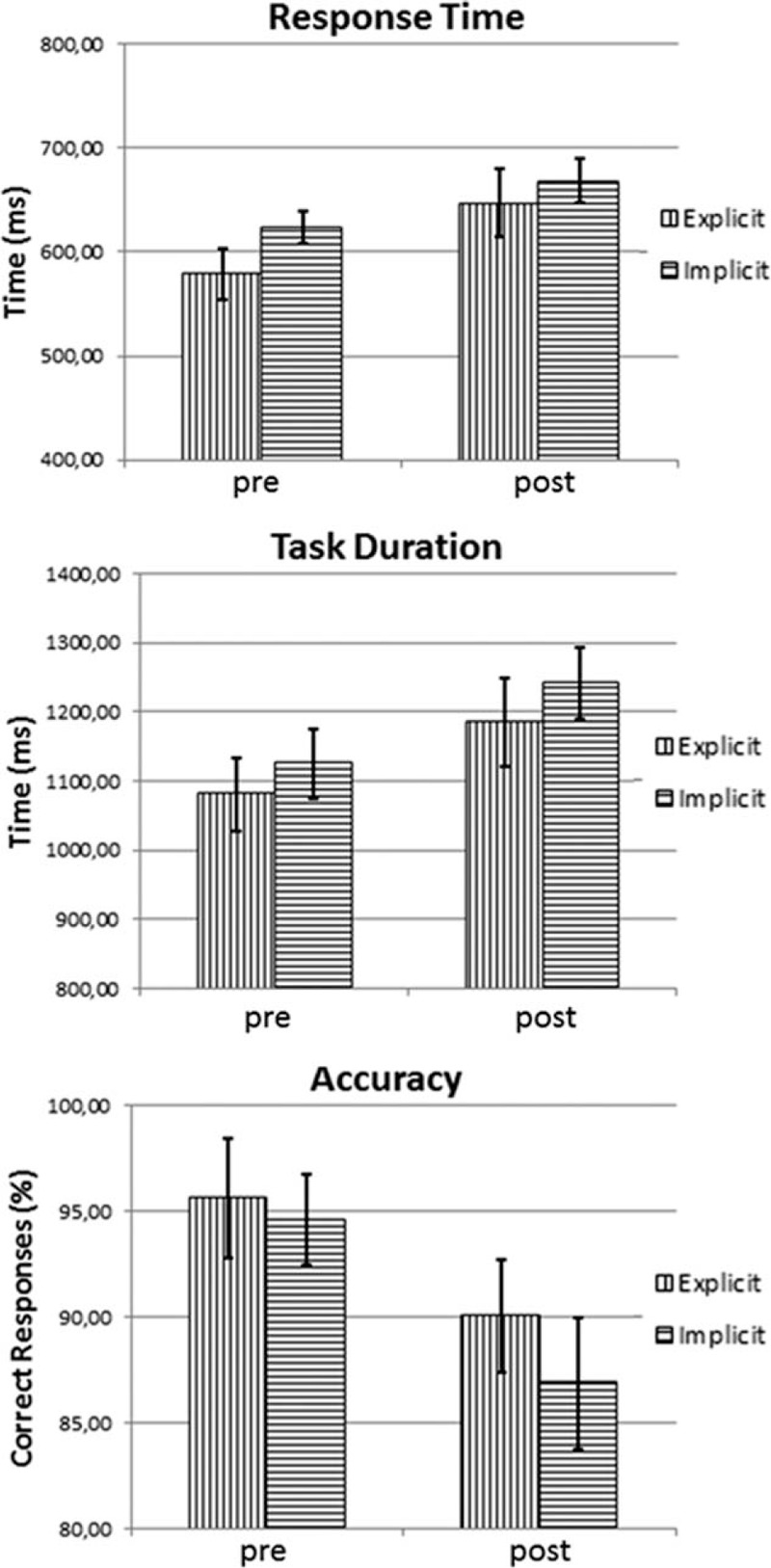
Mean values ( ± SD) of response time (in milliseconds), task duration (in milliseconds), and accuracy (in percent of correct responses) of motor performance in explicit as well as implicit trials. Values were measured before (pre) and at the end (post) of the acute exhaustive exercise. SD = standard deviation.

Figure 4 illustrates the effect of the exhaustive exercise on both explicit and implicit motor trials, in terms of RT, TD, and AC. One-way ANOVA showed significant differences within the displayed samples (*P* value < 0.0001). In particular, as can be seen in Table 1 where are illustrated the results of paired *t* test (2-tailed) of data illustrated in Figure 4, at the end of the exercise there is, in both motor trials, a significant increase of RT and DT as well as a significant worsening of AC. No gender difference was detected for the 3 analyzed parameters (Table 2).

**TABLE 1:**
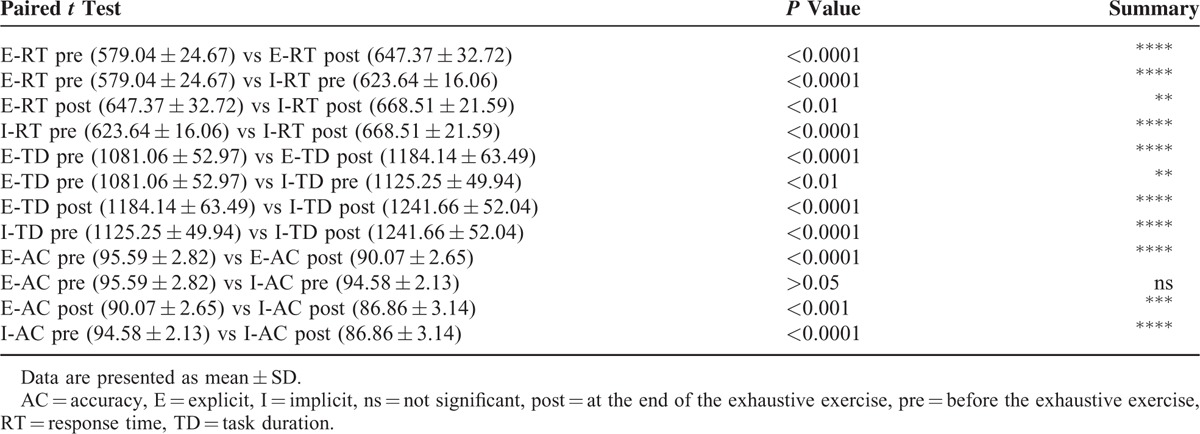
Results of Paired *t* test (2-tailed) of Data Illustrated in Figure 4 (N = 28)

**TABLE 2:**
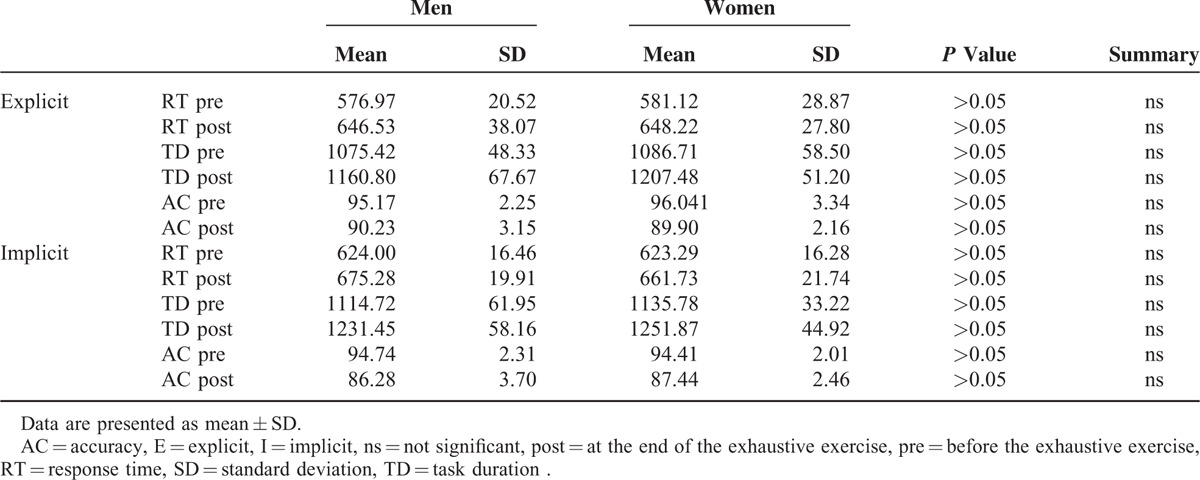
Results of Unpaired *t* Test (2-tailed) of Data Illustrated in Figure 4, Obtained by Separating Men (N = 14) and Women (N = 14)

**FIGURE 5:**
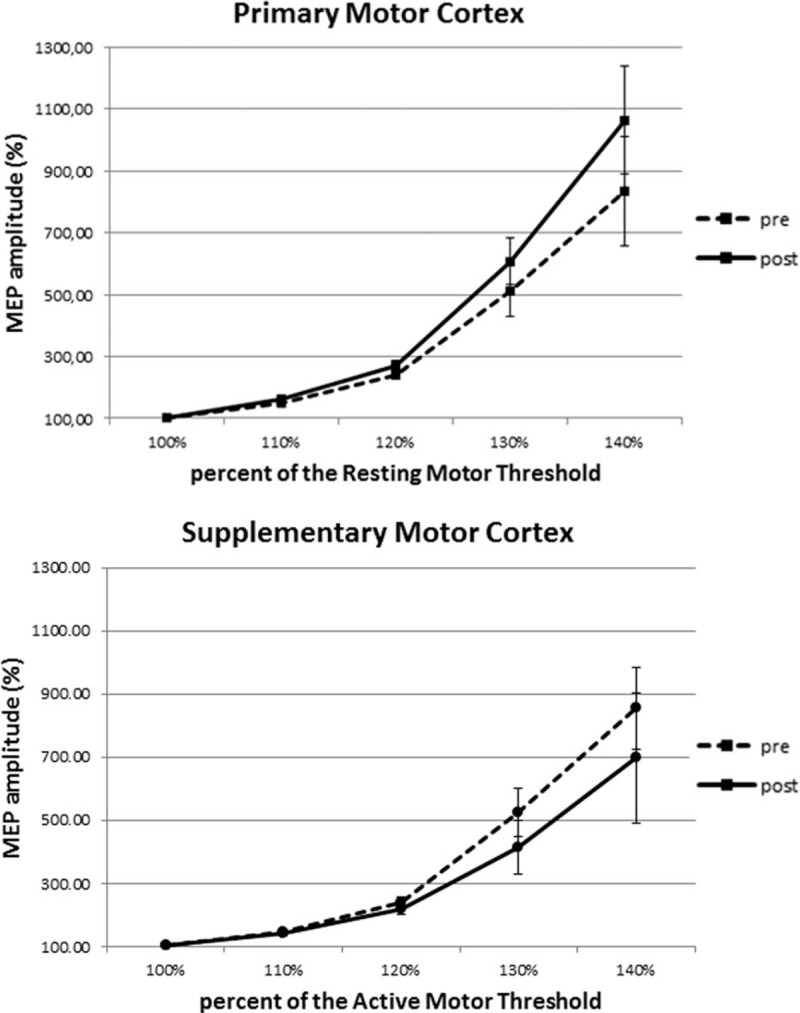
Recruitment curves constructed from the mean amplitudes ( ± SD) of MEPs reported at the 110%, 120%, 130%, and 140% of the resting motor threshold for primary motor area and of the active motor threshold for supplementary motor area. For each cortical area curves obtained before (pre) and at the end (post) of the acute exhaustive exercise are shown. SD = standard deviation.

Recruitment curves in Figure 5 illustrate the changes of excitability of M1 and SMA before and at the end of the exhaustive exercise. As can be seen in Table 3 where are illustrated the results of paired *t* test (2-tailed) of data illustrated in Figure 5, the excitability of M1 was significantly improved at the end of the exercise, whereas that of the SMA was significantly worsened. No differences between men and women were detected in both M1 and SMA (Table 4).

**TABLE 3:**
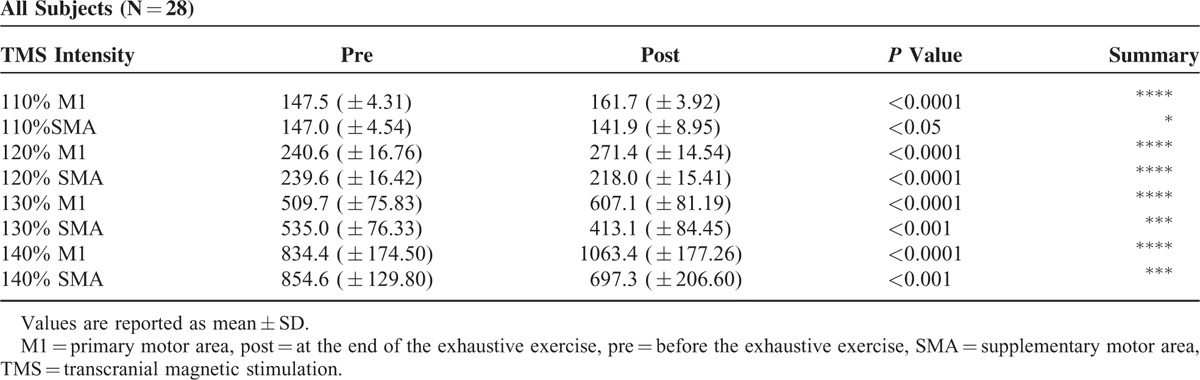
Results of Paired *t* Test (2-Tailed) of Data Illustrated in Figure 5

**TABLE 4:**
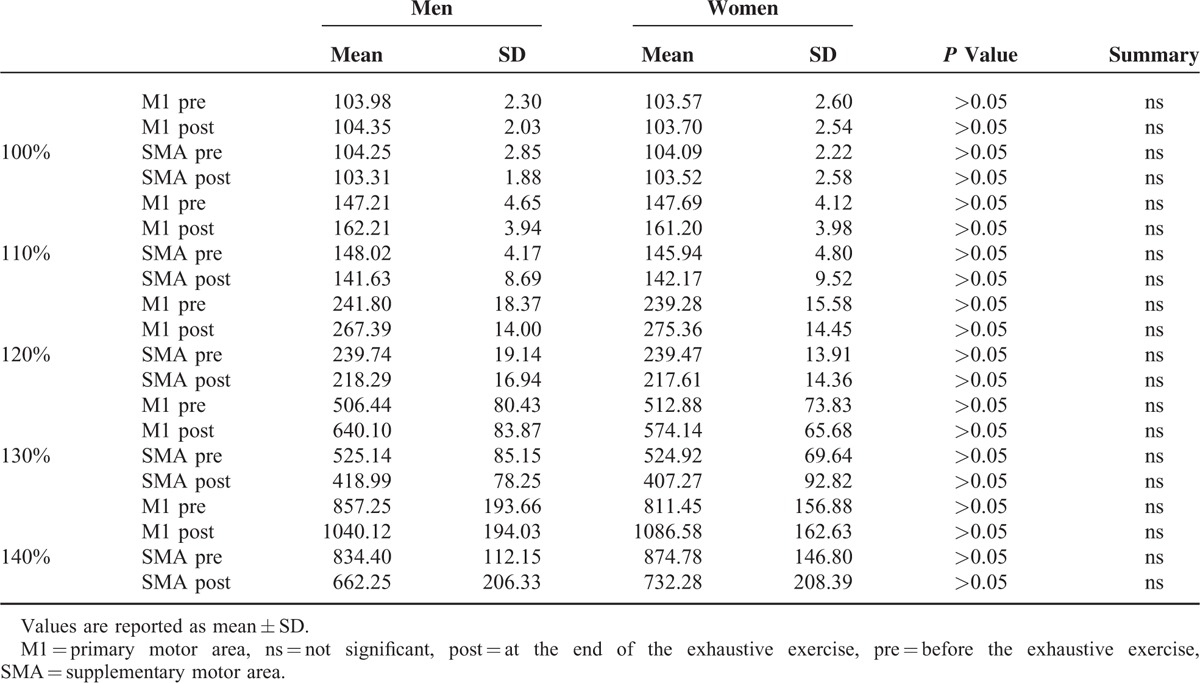
Results of Unpaired *t* Test (2-Tailed) of Data Illustrated in Figure 5, Obtained by Separating Men (N = 14) and Women (N = 14)

## DISCUSSION

In the present study we observed that, after an acute exhaustive exercise, the large increase of blood lactate is associated with a significant worsening of immediate (defined as the explicit trial) as well as delayed (defined as the implicit trial) reproduction of a sequential visuomotor task paradigm, without gender differences. As a matter of fact, we observed that, at the end of the exercise, whose duration did not exceed 5 min, there is an increase of response time and duration of task (i.e., RT and TD) as well as a decrease of correct responses (i.e., AC) in both motor trials. It is worth noting that 7 minutes after the conclusion of the exhaustive exercise, the blood lactate levels remained significantly higher than those measured before the exercise, with a mean value of 6.1 to 6.2 mmol/L.

In the explicit trial the participants were asked to simply reproduce a sequence of finger tapping while continuously watching the sequence order, mainly involving a visuomotor programming component. In the implicit trial the subjects have to use their working memory to subsequently reproduce the same sequence of movements, and the role of working memory in motor learning is well known.^30^ It has been recently observed that at the end of exhaustive exercise, the strong increase of blood lactate is associated with a worsening of the working memory.^31^

It is widely recognized, for example,^32^ that, on motor learning, in addition to the working memory, attention also carries out plays a critical role; in this aspect, it has been demonstrated that an increase of blood lactate, induced by an exhaustive exercise or an intravenous infusion, is associated with a worsening of attentional processes.^7^ Furthermore, it has been observed that,^33^ after a steady 30 minutes aerobic exercise performed at 60% and 80% of maximal oxygen consumption, a worsening of attentional capabilities does not occur, unless there is an increase of blood lactate >4 mmol/L, a classical marker used to define the onset of blood lactate accumulation (OBLA), considered a valid indicator for transition from aerobic to anaerobic performance.^34,35^ Also in equestrian performing show jumping, a deterioration of attention (intensity and selectivity) and a worsening of performance were observed only when blood lactate exceeds OBLA.^36^

In the present experiments, the execution of both visuomotor task paradigms and recruitment curves was carried out in no more than 5 minutes and the levels of blood lactate were still >4 mmol/L (i.e., above OBLA) up to 7 minutes after the end of the exhaustive exercise.

Furthermore, as can be seen in Figure 4 and Table 1, it was observed a more pronounced decay in accuracy in the implicit task, requiring working memory, compared to the explicit task. Therefore, it is reasonable infer that the increase in blood lactate that occurs after an exhaustive exercise may be responsible for a worsening of motor learning, at least as it can worsen neurocognitive capabilities as working memory and attention.

In the present study, we also observed that, at the end of the exhaustive exercise, there is a change of excitability in both M1 and SMA, without differences between men and women. In particular, the excitability of M1 was increased whereas that of SMA decreased. Opposite effects on excitability of adjacent cortical areas lead to exclude that the effects induced by TMS are nonspecific, due, ad example, to a postexercise cortical increase in temperature, acidity, and so on.

It has been suggested that M1 might contribute to motor learning by optimizing the timing of visuomotor processing.^14^ In fact, activation of M1 seems to produce a task-dependent effect on learning and memory formation^15^ and M1 appears to be involved in the early consolidation of motor skills.^16^ Instead, SMA might contribute to the preparation and execution of learned motor sequences;^17^ in this way, SMA seems to play an important role in linking cognition to action.^19^

Several studies have demonstrated that M1 and SMA all appear to be particularly important in the early stage of motor skill acquisition.^22,23^ Kim and Shin^24^ recently compared the effects of 20 Hz repetitive TMS on M1 and SMA, in learning motor skills. Their finding indicated an important role of SMA compared to M1, in implicit motor learning.

Therefore, the present results suggest that an exhaustive exercise, with the associate high blood lactate levels, exerts a positive effect on M1, involved in the early consolidation of motor skills,^16^ but a negative action on SMA, contributing either to the preparation and execution of learned motor sequences^17^ and in implicit motor learning.^24^ Moreover, on the basis of the suggestions of Nachev et al,^19^ it is possible to hypothesize that the high levels of blood lactate induced by a maximal exercise could worsen the role of SMA in linking cognition to action.

Improvement in M1 excitability after a fatiguing exercise has been previously described.^8,37^ However, an unexpected result was that the observed facilitation of M1 excitability does not show significant differences between men and women. This is in contrast with previous results showing that enhancement of excitability of M1, concomitant with an increase of blood lactate, is significantly greater in women than in men.^38^ It could be argued that this discrepancy could be dependent on the fact that the women of that study, in order to standardize the effects of sex hormones on cortical excitability, were submitted to TMS of M1 in the midluteal phase of menstrual cycle. In the present research, because of the timing of the experimental protocol, the women who voluntarily participated were studied independently of the phase of their menstrual cycle.

In conclusion, an increase of blood lactate, as that occurring at the end of an acute exhaustive exercise, is associated with various effects on the frontal lobe, as a worsening of attentional processes,^7^ an improvement of excitability of M1 and a worsening of the excitability of SMA. Furthermore, experiment carried out with visual^11^ and somatosensory-evoked potentials^12^ have shown that increases of blood lactate are associated, in both cases, with an improvement in the conduction time between the periphery and the primary sensory areas and with the worsening of intracortical communication between the primary areas and additional cortical regions. Moreover, different effects induced by lactate have been observed *in vitro* on neurons and astrocytes.^13^

In this way, high blood lactate levels appear to exert in the brain a dual action, with a protective effect, at least at level of primary cortical areas (as M1, striate cortex, or primary somatosensory cortex), although at the expense of efficiency of adjacent areas, as SMA.
